# Viratrum Veride

**Published:** 1856-07

**Authors:** Isham H. Ragan

**Affiliations:** Lithonia, Ga.


### ARTICLE II.

*Viratrum Veride.* By ISHAM H. RAGAN, M. D., of Lithonia, Ga.

We are aware that there has been a great deal written upon this medicine, since the discovery of its sedative properties by Dr. Norwood; but for the last two years, it seems to have been but little noticed in medical periodicals, and we feel confident that the excitement died away before its merits were unanimously acknowledged, even among the physicians of the South; therefore we think an article from a more able pen than ours would not be inappropriate.

We are not prepared, from our experience, to grant all that has been claimed for viratrum veride, yet we have seen enough to convince us that it is entitled to rank among the best of medicines. Some physicians seldom if ever use it, from the fact, as they say, that it is uncertain and dangerous. When used appropriately, we think we have no medicine more apt to produce the effect for which it is given than viratrum veride. There is no practitioner, I presume, who has not been disappointed, to some extent, in the administration of medicines, yet he does not exclude every article that fails in some instances to produce precisely the effect anticipated. It is said, that it may be given to produce its specific effect and yet the disease go on and prove fatal. This is very true; but we must remember that disease does not always yield, even to the most appropriate remedies. As to its being dangerous, we are fully aware that, like all other active remedies, it should be judiciously and cautiously used. Yet we do not think it so dangerous that physicians should hesitate to use it where it is indicated. We are sure we have never seen the evil overbalance the benefit derived from it in any appropriate case. Nor do we now remember to have ever seen any alarming symptoms developed from its administration. There is another barrier to its general use which no doubt causes some practitioners to lay it aside and object to its employment; some for want of confidence in their own judgment, and others seeking popularity. This barrier is the prejudice that exists in the minds of some people against its use. This difficulty is, of course, very embarrassing to the physician, yet, he who is true to the cause of humanity should labor to convince the common people of the errors, notwithstanding the task is fraught with difficulties, and not seek to establish himself in a community by endorsing their ereneous notions respecting medicine, which we trust no regular physician does.

The ideas we may offer upon the use of the viratrum veride may not be concurred in by all who may read this article. But we proceed upon the ground that every man has a right to give his opinion deduced from his own experience, and is also entitled to the privilege of examining the opinions of others, and retaining the good and rejecting the worthless.

The disease in which we have found the viratrum veride most beneficial, are those of an inflammatory nature. In these diseases we are of the opinion that it is far more effectual and less objectionable than any other medicine we have ever used. Tartar emetic has previously stood at the head of the list of remedies used in pneumonia, &c., and has been a valuable medicine in that and other affections. We cannot assert, as some of the advocates of viratrum veride have done, that without it we could not practice medicine, but in its absence, would resort to tartar emetic with a good deal of confidence; still with far less than we now resort to the use of viratrum veride. It does, without doubt, reduce the action of the heart and arteries more effectually than tartar emetic. We have never been able, in a bad case of pneumonia, to reduce the pulse to a natural standard with tartar emetic, but with viratrum veride we can, easily, reduce them to that, and lower if desirable. The nauseating effects of tartar emetic, when used to procure its best action upon the disease, are very disagreable to the patient. In the use of viratrum veride, these are entirely avoided after the first few doses. When the system is once under its influence you may then say to the pulse "so fast shalt thou beat and no faster," and yet keep your patient entirely comfortable. It does not effect the bowels as full doses of tartar emetic do, which is a matter of no little importance, as to be able to control the bowels is an object very desirable to the physician. Then if you can effect the same object with viratrum veride more easily and with less annoyance to your patient, why not use it in preference to other articles? If a diminution of the action of the heart and arteries be the object for which we use either, we see from the above that viratrum veride is without doubt the best remedy. That this is the main object, we presume, is generally agreed. Some may contend that tartar emetic acts as a revulsive by irritating the stomach and bowels; but we would prefer external counter-irritation. According to our experience, the less the tartar emetic effects the bowels the greater is the benefit derived. By lessening the heart's action, we diminish the quantity of blood flowing to the inflamed part, and by this means give nature, or the *vis medicatrix naturæ*, a better chance of relieving the diseased organ. And we think, in order to render the greatest assistance to nature, we should reduce the pulse to about the natural standard. By reducing them still lower we may stop the excessive flow of blood to the part, and entirely, or in a great measure, check the progress of the inflammation. But may not the heart beat too slow for the *vis medicatrix naturæ* to exercise its healing powers? May we not expect the effused and extravasated matter to be thrown off more easily when the circulation is at its natural standard? We are inclined to answer in the affirmative. In the first stage of inflammatory diseases where the only indication is to relieve the excitement, we think viratrum veride is sufficient for that purpose, and this is the principal benefit we expect to derive from its use. In the more advanced stages of inflammation where nature seems to need assistance to heal the inflamed organ, even after the excitement has been subdued, we then resort to remedies necessary to attain this end, such as blistering, poulticing, &c., and use the viratrum veride merely to subdue the excitement. For we do not conceive that viratrum veride supersedes the necessity of blistering, any more than tartar emetic, only so far as blistering changes the force of the circulation from one part to another.

We have used the viratrum veride in the incipient stages of pneumonia, and have seen the disease removed almost like a magic. At a period a little more advanced we have used it and have witnessed the gradual disappeasance of the affection without any unpleasant consequences; and in still more advanced stages of this diseases we have used it in connection with other remedies with very happy results. And again, we have used it and seen it fail, as we, occasionally, see all other remedies fail. We have also used it in other diseases, such as bronchitis, puerperal fever, &c., with entire satisfaction. And in typhoid fever complicated with pneumonia, we know of no medicine half so valuable. In fact, before it came into use, we were compelled to depend almost entirely upon blisters, fomentations, opiates, &c., as all other remedies, commonly used in pneumonia were contraindicated by the typhoid symptoms. But now, by using the viratrum veride in connection with blisters, &c., we can relieve many cases, which, in our humble opinion, would inevitably be lost. It is said to be an excellent remedy in typhoid fever generally, but we have not found it very beneficial in the simple form of this fever. But in that form, which we think may be very appropriately termed inflammatory typhoid fever, that is, where there seems to be no local inflammation, but where the pulse and symptoms generally indicate a tendency to inflammation, we think the viratrum veride subdues those inflammatory symptoms, and prevents local disease, which would be very likely to set in at some time in the course of the fever, were these symptoms to remain unchecked. Then the physician who judiciously uses the viratrum veride as an arterial sedative, and at the same time expects no other very important result from it, and also uses other remedies to meet other indications, we think will not be disappointed, but will find it a valuable medicine in the treatment of disease of an inflammatory nature.

